# Coexistence mechanisms at multiple scales in mosquito assemblages

**DOI:** 10.1186/s12898-014-0030-8

**Published:** 2014-11-11

**Authors:** Gabriel Zorello Laporta, Maria Anice Mureb Sallum

**Affiliations:** Departamento de Epidemiologia, Faculdade de Saúde Pública (FSP), Universidade de São Paulo (USP), São Paulo, SP Brazil; Laboratório de Informática Médica (LIM/01), Faculdade de Medicina (FM), Universidade de São Paulo (USP), São Paulo, SP Brazil

**Keywords:** Biodiversity, Biotic interactions, Coexistence, Community, Ecology, Mosquitoes, Resource partitioning, Tropical rainforest

## Abstract

**Background:**

Species coexistence in mosquito assemblages may depend on mechanisms related to interspecific resource partitioning occurring at multiple scales. In the present work we investigated co-occurrence or spatial segregation in mosquito assemblages sharing resources at micro-habitat, habitat and landscape scales. Environmental characteristics, mosquito fauna as adults and larvae were assessed along vegetation gradient in a natural landscape of tropical rainforest. Huisman-Olff-Fresco (HOF) and Generalized Additive (GAM) models were employed to explore relationships between abundances of potential competitors in mosquito assemblages and vegetation gradient (e.g., scrublands, mixed arboreal vegetation and dense ombrophilous forest). We tested hypotheses concerning mosquito species co-occurrence or spatial segregation employing binomial logistic regression models.

**Results:**

Co-occurrences and spatial segregation of mosquito species showed evidences of three scales of coexistence mechanisms: 1) micro-habitat - scale 1: different behaviors in response to food availability in specific vertical strata within larval container; 2) habitat - scale 2: specialized strategies related to heterogeneity of resource availability among larval containers and 3) landscape - scale 3: asymmetrical competition dependent upon the context of abiotic and biotic variables.

**Conclusion:**

Results of the present work suggest that coexistence mechanisms can concomitantly work at multiple scales.

**Electronic supplementary material:**

The online version of this article (doi:10.1186/s12898-014-0030-8) contains supplementary material, which is available to authorized users.

## Background

One of the approaches that has shown to be effective in the analysis of multi-species assemblages is to consider resource utilization pattern data [1-3]. The examination of resource utilization patterns reveals aspects of species coexistence in an ecological community [4-6]. The degree of spatial overlap in resource use is an important parameter to explain community organization and the pattern of species coexistence [7-9]. Interspecific resource competition within larval containers (e.g., bromeliads, tree holes or man-made vessels), all of which are food-limiting environments, results in the simultaneous presence of multiple mosquito species. For the bromeliad-dwelling mosquito community, Gilbert et al. [10] proposed two coexistence mechanisms among mosquitoes. One provides co-occurrence of species within a given larval container via specialization of feeding behavior, while the other performs spatial segregation of species with overlapping use of resource by specializing them at different bromeliad sizes. The third mechanism of coexistence was assessed from the best studied pair of competitors, *Aedes albopictus* and *Aedes aegypti*, regarding the asymmetrical competition, which is significantly associated with the diversity of resources in a landscape scale [11,12].

Species coexistence may occur in heterogeneous environments by spatial storage effect: covariance between the environment and competition [13,14]. For container-dwelling mosquitoes, different container types may represent a tradeoff for mosquito larvae between the risk of dry out and presence of large predators [15,16]. The differences in the species responses to this kind of conflicting pressures affect the competitive outcomes at different bromeliads. As the distribution and abundances of bromeliads and other larval sites vary in space and time, spatial heterogeneity may promote coexistence by changing the scale in which competing species coexists [10,17].

**Figure 1:**
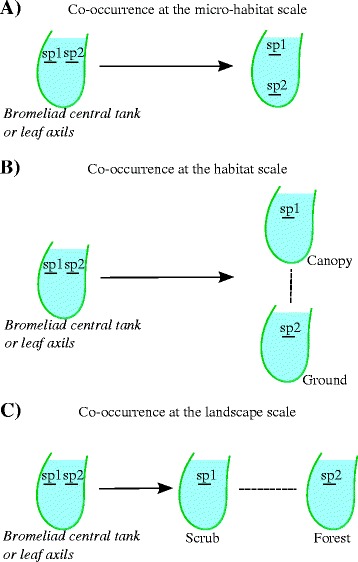
**Coexistence mechanisms. A)** Larva of the species 1 (sp1) and larva of the species 2 (sp2) can co-occur at the micro-habitat (e.g., bromeliad central tank or leaf axils) if they can vertically partition food and space (i.e., one feeds at the surface and the other at the bottom). **B)** The coexistence among larvae of competitor species is mediated by vegetation heterogeneity that allows occurrence of the sp1 in the ground (e.g., terrestrial bromeliads) and the sp2 in the canopy (epiphyte). **C)** Along a physiognomic gradient of vegetation, interactions among potential competitors (sp1 and sp2) are determined by the landscape context, causing one species to be superior competitor in a physiological stressful scenario (scrub) and the other as being an efficient competitor in the forest.

Here we focus on host-seeking adult females and larvae of mosquito species across a gradient of vegetation physiognomies in a natural landscape of Atlantic rainforest. Previous research on mosquito communities has provided evidence for coexistence mechanisms within the scope of container-dwelling mosquitoes. Herein, coexistence mechanisms in the whole mosquito community, from species occupying ground waters to other that occupy natural containers, are considered at micro-habitat, habitat and landscape scales. Moreover, most of the mosquito surveys performed in areas of tropical rainforest in southeastern Brazil was focused on testing hypotheses concerning particular mosquito fauna along a gradient of anthropogenic modifications [18]. This approach has led to baseline knowledge about mosquito species’ responses under the influences of human-caused changes in ecosystems, reinforcing the importance of synanthropy (i.e., the ability of a mosquito species to use resources in a man-made environment [19]). Interpreting results through the lens of ecosystem changes, these studies left gaps in the knowledge about properties of natural and undisturbed mosquito communities in tropical rainforest. Our approach is to fill some of these gaps, by testing hypotheses about mosquito species coexistence. More specifically we were interested in investigating co-occurrences of potential competitors at the micro-habitat scale (Figure 1A) [10,20], habitat scale (Figure 1B) [10,17] and landscape scale (Figure 1C) [12].

## Methods

### Study system

**Figure 2:**
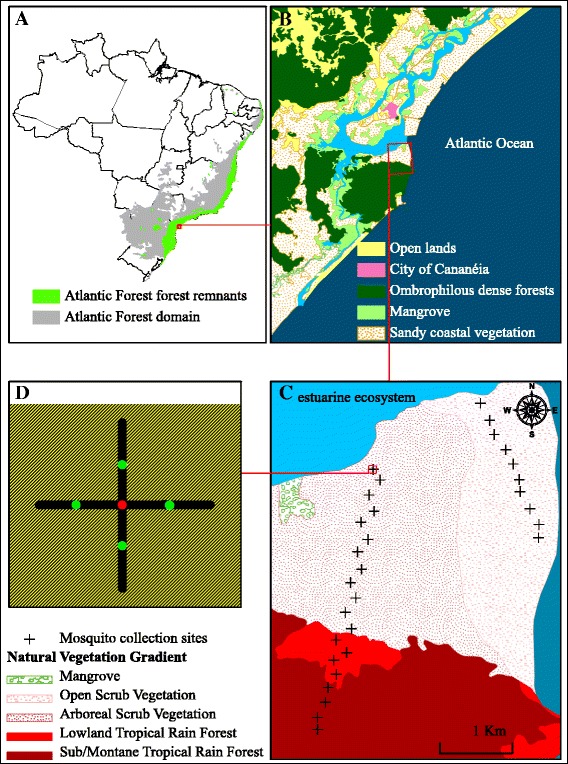
**Study area. A)** The Atlantic Forest domain and its forest remnants. **B)** Atlantic Forest remnants in São Paulo and Paraná States. **C)** Location of the Parque Estadual da Ilha do Cardoso (Bernardi et al. [22]). **D)** Collection sites for measurements of vegetation complexity, the number of ground and phytotelmata oviposition sites and the number and identification of immature and adult mosquitoes. Source: Conservation International (CI), Critical Ecosystem Partnership Funding (CEPF), Instituto Nacional de Pesquisas Espaciais (INPE).

The Brazilian Atlantic Forest is a tropical biome with mountainous forests and seasonally flooded coastal ecosystems [21]. Within the Atlantic Forest domain, regions of the coastal plain contain forest remnants (Figure 2A) that include dense ombrophilous forests intermixed with mangroves, dune ecosystems and coastal islands (Figure 2B). Bernardi et al. [22] measured abiotic and biotic variables and applied remote sensing techniques to produce a thematic map of the vegetation types in the Parque Estadual da Ilha do Cardoso (PEIC) (Figure 2C). These authors also indicated that physiological stress factors tend to decrease along the physiognomic gradient, with inland forests containing high levels of above ground biomass and soil nutrients. Studying bromeliad food webs in an understory of mixed arboreal vegetation in the PEIC, Romero and Srivastava [16] found a diverse arthropod fauna composed of aquatic insect larvae of several functional groups. The functional group of filter feeders is represented by the mosquito community in which the larvae assemblage, composed of different species, may experience interspecific resource competition.

### Sampling design

**Figure 3:**
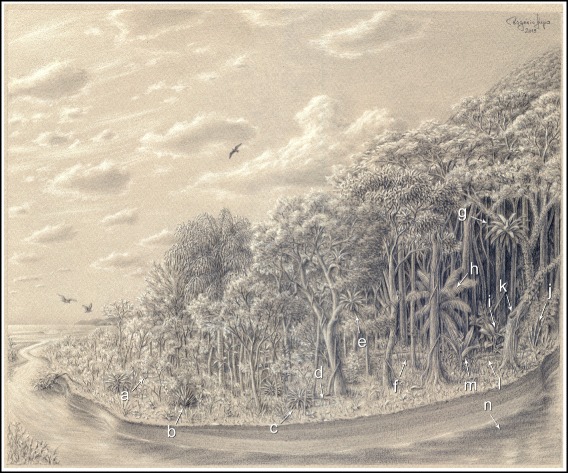
**Mosquito larvae collections across the gradient of forest physiognomies.** This drawing represents the vegetation complexity and mosquito larvae natural containers and ground waters which varied accordingly as follows: a) ephemeral ground pool; b) terrestrial bromeliad plant (*Quesnelia* sp.); c) terrestrial bromeliad plant (*Nidularium* sp.); d) ephemeral ground pool; e) epiphytic bromeliad plant (*Vriesea* sp.); f) ephemeral ground pool; g) epiphytic bromeliad plant (*Vriesea* sp.); h) leaf axils in palm tree (*Euterpe* sp.); i) leaf axils in phytotelmata (*Calathea* sp.); j) hollow internode of bamboo plant (*Merostachys* sp.); k) tree hole in long-lived tree; l) ephemeral ground pool; m) flower bracts in phytotelmata (*Heliconia* sp.) and n) margins of permanent lotic waters.

Mosquito assemblages were assessed from 30 collection sites that spanned physiognomies varying from scrub vegetation to dense forest in the PEIC (25° 06’ S 47° 53’ W, Figure 2C). Each collection site was composed of a cross consisting of four 15-m long transects (Figure 2D). Previous long-term field collections conducted in the southeastern Atlantic Forest at the end of a hot and rainy summer showed that mosquito densities were high and that microclimatic factors were favorable for maintaining high levels of diversity in mosquito assemblages [23]. Given those findings, we sampled mosquitoes over 20 days in both March 2009 and April 2010. The data were aggregated across years in subsequent analyses. Adults were collected at the middle (red dot) in Figure 2D using automatic CDC-CO_2_ as described by Laporta and Sallum [24] (from 06:00 to 18:00 h) and CDC-light traps (from 18:00 to 06:00 h) over a 24-hour sampling period. Each CDC trap was set 1.5 m above ground level to attract both canopy and ground mosquitoes [25]. Measures of vegetation complexity and its heterogeneity were assessed at the middle of each transect (green dots in Figure 2D). The numbers of phytotelmata and ground pools were counted in each transect (1-m wide). Immature specimens were sampled in phytotelmata and ground pools (Figure 3).

Adult mosquitoes collected in the field were maintained inside collection vials with silica to prevent fungal growth and damage to the specimens. Mosquito larvae were maintained in the laboratory to obtain males and females associated with both larval and pupal exuviae when possible. Species were identified based on external morphological characters using identification keys [26-29]. Mosquito nomenclature followed Knight and Stone [30]. Larvae identifications were undertaken using procedures adopted in Marques et al. [31].

The complexity of vegetation gradient was assessed by 4 measurements taken in each collection site (see green dots in Figure 2D). Data from vegetation complexity was obtained following the protocol described by Pardini et al. [32]. Accordingly, foliage density (i.e., vertical length of plant leaves) was measured for plants of each of the five forest strata 0-1, 1-5, 5-10, 10-15, >15 meters above the ground. The length of plant leaves was measured, along a vertical column, with a laser rangefinder Bosch™ DLE50 (Bosch™, Stuttgart, Germany) by considering a vertical line from the bottom of the plant to the top of the leaves when straightened. A 4-meter pole was also utilized to help to establish a vertical line. Foliage density values were estimated for each stratum and for each transect in all collection sites. A principal component analysis (PCA) on the values of foliage density for all strata and for all transects was conducted. The inter-transect mean of eigenvalues of the first axis was estimated for each collection site to represent the vegetation complexity. The coefficient of variation of vegetation complexity was estimated and then it was utilized as indicative of heterogeneity of vegetation complexity.

The present research was certificated with the authorization by the Instituto Brasileiro do Meio Ambiente e dos Recursos Naturais Renováveis (IBAMA) of the Brazilian Ministry of Natural Environment (13136-1, 13136-2).

### Data analysis

Response patterns for the distribution of mosquito species as adults were analyzed as a function of the vegetation complexity. The response patterns of potential competitors were used as exploratory analysis of species co-occurrences or spatial segregation. A model selection approach was applied using Huisman-Olff-Fresco (HOF) models and extensions as described by Jansen and Oksanen [33] as follow: I (flat) - there was no response pattern along the vegetation complexity; II (monotone) - the response pattern was linearly correlated with the vegetation complexity; III (plateau) - the response pattern was partially linear and partially asymptotic; IV (symmetric) - the response pattern was similar to a bell-shaped response curve; V (skewed) - the response pattern was a skewed non-monotonic response curve; VI (symmetric bimodal) - the response pattern had a symmetric bimodal shape; VII (skewed bimodal) - the response pattern had a bimodal shape with two skewed maxima.

Five model parameters, *a*-*d* and *f*, were optimised during the process of model fitting. The number of parameters decreased with increasing model simplicity: Model VII had five parameters (*a*, *b*, *c*, *d*, *f*), Models VI and V had four parameters (*a*, *b*, *c*, *d*), Models IV and III had three parameters (*a*, *b*, *c*), Model II had two parameters (*a*, *b*) and Model I had one parameter (*a*). The process of model fitting for the HOF models and their extensions was based on the maximisation of a log-likelihood function using iterative non-linear methods to estimate parameters *a*-*d* and *f*. The process of model fitting was applied with the *eHOF* package in the R computational environment 3.0.0 [33]. The model selection approach was based on the Akaike Information Criteria corrected for small samples (AICc). The best model was selected as that associated with the smallest AIC value, and a value of evidence of ∆AICc >2 was adopted to differentiate plausible models. A value of evidence of ∆AICc >2 is equivalent to three consecutive successes in a Bernoulli independent trial. Because candidate models can adjust similarly to the data, we also considered functional redundancy, defined as the presence of both simple and complex models with similar response patterns [34].

Co-occurrences or spatial segregation of mosquito species as larvae were evaluated in micro-habitat, habitat and landscape scales in order to determine evidences of species coexistence. Thus, logistic binary regression models were employed in order to fit biological data within R 3.0.0 programming environment with the aid of *epicalc* package [35]. The logistic binary regression model was applied such that species presence-absence was fitted with another species presence-absence in a specific set of bromeliads or ephemeral ground pools. Odds ratio (OR) was estimated in order to assess associations between the two potential competitor species, under the null hypothesis OR =1 (there is no association). An OR statistically higher than 1 (OR >1) provides evidence for species coexistence in micro-habitat scale. For an OR lower than 1 (OR <1), species coexistence may occur either at habitat or landscape scales.

In order to see overall responses from a community perspective, HOF model selection was employed to determine whether species richness response patterns varied monotonically or non-monotonically against vegetation gradient. Furthermore, the coefficient of variance of vegetation structure, i.e., the square root of inter-transect variance divided by inter-transect mean of eigenvalues of the first axis of PCA, was estimated and then it was utilized as indicative of heterogeneity of vegetation structure, according to Chaves et al. [17]. HOF model selection was again employed to determine whether heterogeneity of vegetation structure can be the underlying mechanism that influences patterns of diversity (richness and evenness-dominance) of the mosquito fauna.

Finally, 95% Confidence Intervals (95%CI) were calculated by applying GAM (Generalized Additive Models) with the employ of *mgcv* package in the R 3.0.0 program [33].

## Results

After 720 hours of automatic trap sampling effort, 11,660 adult specimens (73 species) were collected from the mosquito assemblages. *Coquillettidia chrysonotum* was the most abundant species (8,081; 69%). Other species were, for example, *Aedes scapularis* (634; 5%), *Anopheles cruzii* (268; 2%), *Wyeomyia quasilongirostris* (258; 2%), *Aedes serratus* (243; 2%), *Wyeomyia muehlensi* (155; 1%), *Culex imitator* (73; <1%) and *Anopheles bellator* (45; <1%). The sample also contained rare species, a total of 16 singletons. No specimens of *Anopheles* (*Nyssorhynchus*) or synanthropic species (e.g. *Aedes aegypti*, *Culex quinquefasciatus*) were recorded.

Mosquito larvae were collected from natural containers and ground waters according to Figure 3. Although it was possible to collect *Wyeomyia aporonoma* and *Sabethes intermedius* from a tree hole in *Ficus* spp. in the forest, most specimens were obtained from bromeliads and ground pools. An average of three bromeliads and one ground pool per site were searched for the presence of mosquito larvae, totaling 98 bromeliads and 30 ground pools. Mosquito fauna from permanent ground pools with emergent vegetation was not sampled because it is supposed to be less controlled by interspecific resource competition than that in small pools, thus larvae of *Cq. chrysonotum* and *Uranotaenia* were not recorded. In the herein study site, permanent ground pools were present along margins of the river that encounters the sea on the plain coast (Figure 3n). Instead, temporary ground pools (Figure 3a, d, f, l) were selected and provided mostly larvae of *Aedes* (*Ochlerotatus*), *Psorophora* and *Culex* (*Melanoconion*). Both terrestrial and epiphytic bromeliads occurred along the gradient, terrestrial more common in scrublands (Figure 3b) and epiphytic bromeliads in the forest (Figure 3g). In the middle of the gradient where mixed arboreal and scrub vegetation occurred, both terrestrial (Figure 3c) and epiphytic (Figure 3e) bromeliads were found, mainly in the genera *Quesnelia*, *Vriesea* and *Nidularium*. Common mosquito inhabitants were *Anopheles* (*Kerteszia*), *Culex* (*Microculex*) and *Wyeomyia* (*Phoniomyia*). In total, 936 specimens were collected as larvae. Species that could be identified to species level were *Ae. scapularis* (n =54), *Ae. serratus* (n =48), *An. bellator* (n =83), *An. cruzii* (n =177), *Cx. imitator* (=96), *Wy. muhelensi* (n =67) and *Wy. quasilongirostris* (n =118).

Results of the PCA on the values of foliage density showed that the first component explained approximately 45.8% of the variation, with the highest importance associated with foliage densities in the strata that were >15 m, 10-15 m and 1-5 m above ground level. This component was used as the main component of vegetation complexity. On scrublands foliage density in the lower strata increased, whereas foliage density in the lower strata decreased in multi-layered forest physiognomies (i.e., dense ombrophilous forests). Vegetation complexities in scrublands and dense forests did not change very much, the former being composed of scrub and the latter by a multi-layer forest. In the middle of the gradient, however, vegetation complexities were a heterogeneous mix of arboreal and scrub vegetation presenting an irregular and spatially discontinuous mosaic of trees and bushes (Figure 3, Additional file 1: Table S1).

**Figure 4:**
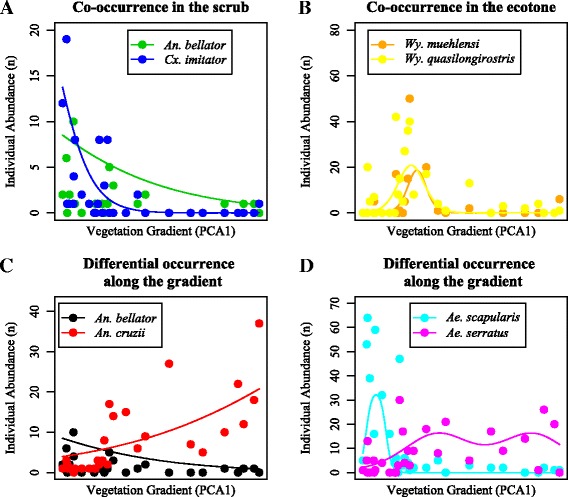
**Observed pattern of co-occurrences or spatial segregation among potential competitors. A)** *An. bellator* and *Cx. imitator* co-occurred on scrublands at habitat scale. **B)** *Wy. muehlensi* and *Wy. quasilongirostris* co-occurred in the transition zone at habitat scale. **C)** *An. bellator* and *An. cruzii* and **D)** *Ae. scapularis* and *Ae. serratus* co-occurred at landscape scale and spatially segregated at habitat scale. GAM for non-monotonic HOF models and 95%CI are in Additional file 7: Figure S5, Additional file 9: Figure S7.

The relationships among mosquito potential competitor species as adults and vegetation gradient varied substantially, showing that some species could co-occur at habitat scale (Figure 4A, B) and others that co-occurred at landscape scale (Figure 4C, D) (see Additional file 2: Table S2, Additional file 3: Table S3, Additional file 4: Figure S1-S4). Co-occurrences and spatial segregation of mosquito potential competitor species as larvae showed evidences of coexistence mechanisms at three scales: micro-habitat, habitat and landscape (Table 1). Abundance distributions of *An. bellator* and *Cx. imitator* were associated with scrublands (Figure 4A). In the micro-habitat on scrublands, the co-occurrence of *An. bellator* and *Cx. imitator* in 41 bromeliads (out of 98) was statistically significant (Table 1). Abundance distributions of *Wy. muhelensis* and *Wy. quasilongirostris* were associated with the arboreal scrub vegetation (Figure 4B). Although *Wy. quasilongirostris* and *Wy. muhelensis* could occur in the same habitat (Figure 4B), they did not at micro-habitat scale and occurred together only in 11 bromeliads, with the former species mostly found in epiphytic bromeliads and the latter species in terrestrial bromeliads. The two other pairs of species, *An. bellator*-*An. cruzii* and *Ae. scapularis*-*Ae. serratus*, could only co-occur on a landscape scale (Figure 4C, D). Abundance distribution of *An. bellator* was complementary to the abundance distribution of *An. cruzii* (Figure 4C) and larvae of *An. bellator* and *An. cruzii* did not frequently co-occur (Table 1). Abundance distribution of *Ae. scapularis* was associated with scrubland, whereas that of *Ae. serratus* had two peaks, one at mid-gradient and the other in forest (Figure 4D). Of the 30 ground pools examined, *Ae. scapularis* and *Ae. serratus* as larvae co-occurred only in 6, and did not co-occur in 22 (Additional file 5: Table S4).

**Table 1:** **Co-occurrences or spatial segregation of mosquito potential competitor species as larvae, Parque Estadual da Ilha do Cardoso, 2009-2010**

		**Logistic regression output**				
**Pairs of species**	**Type of containers/ground waters**	***OR*** **(CI 95%)**	**P (Wald)**	**Pattern**	**Support**	**Process**
*An. bellator* - *Cx. imitator*	bromeliad (Figure 3b,c)	8.75 (3.47, 22.04)^a^	< 0.001	Co-occurrence at micro-habitat scale (e.g., bromeliad)	Gilbert et al. [10], Yee et al. [20] and Marques et al. [31]	Specializing feeding behavior within a given larval container
*Wy. muehlensi - Wy. quasilongirostris*	bromeliad (Figure 3c,e)	0.15 (0.06, 0.37)^b^	< 0.001	Co-occurrence at habitat scale (e.g., ecotone); spatial segregation at micro-habitat	Gilbert et al. [10] and Chaves et al. [17]	Spatial heterogeneity promote coexistence by changing the scale at which competing species can coexist
*An. bellator* - *An. cruzii*	bromeliad (Figure 3b,c,e,g)	0.14 (0.05, 0.34)^b^	< 0.001	Co-occurrence at landscape scale; spatial segregation at micro-habitat (e.g., bromeliads) and habitat scales	Juliano [11,12]	Biotic interactions involving mosquito larvae are modulated by effects of context dependence across habitat gradients
*Ae. scapularis* - *Ae. serratus*	ephemeral ground pools (Figure 3a,d,f,l)	0.1 (0.02, 0.61)^b^	= 0.013	Co-occurrence at landscape scale; spatial segregation at micro-habitat (e.g., ephemeral pools) and habitat scales	Juliano [11,12]	Biotic interactions involving mosquito larvae are modulated by effects of context dependence across habitat gradients

^**a**^: this is a significant result under the null hypothesis *OR* =1. Thus there is co-occurrence between the two species.

^**b**^: this is a significant result under the null hypothesis *OR* =1. Thus the two species do not co-occur.

**Figure 5:**
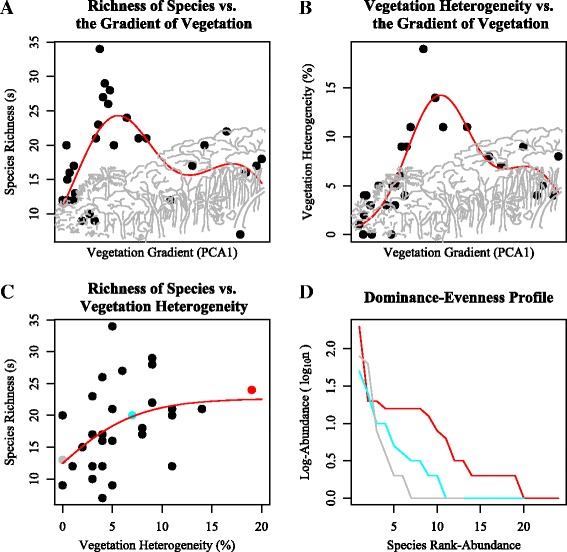
**Relationships between mosquito diversity and the gradient of forest physiognomies. A)** Mosquito species richness has a bimodal, skewed response pattern. The strong peak is related to the heterogeneity of vegetation complexity. **B)** The heterogeneity of vegetation complexity has also a bimodal skewed response pattern, similar to mosquito species richness. **C)** Mosquito species richness increased linearly with heterogeneity of vegetation complexity until it reached an asymptote where a mix of arboreal and scrub vegetation occurred. **D)** The mosquito assemblage evenness-dominance profile as a function of heterogeneity of vegetation complexity. High levels of heterogeneity of vegetation complexity resulted in assemblage evenness, whereas medium to low levels of heterogeneity of vegetation complexity resulted in the presence of dominant assemblages. GAM for non-monotonic HOF models and 95%CI are in Additional file 8: Figure S6.

The association between mosquito species richness and vegetation gradient had a bimodal hump-shaped pattern (Figure 5A, Additional file 6: Table S5). The vegetation heterogeneity also showed a bimodal hump-shaped pattern related to vegetation gradient (Figure 5B). Mosquito species richness had a plateau correlation with vegetation gradient, i.e., it linearly increased up to a plateau (Figure 5C). The increase of vegetation heterogeneity was also associated with the rise of evenness (Figure 5D).

Analyses performed with GAM, a form-free method, detected the same overall pattern yielded with the employ of HOF models, i.e., a form-imposed function (see Additional file 7: Figure S5, Additional file 8: Figure S6 and Additional file 9: Figure S7).

## Discussion

Co-occurrences and spatial segregation of mosquito species were assessed at micro-habitat, habitat and landscape scales along vegetation gradient in a natural and undisturbed landscape of tropical rainforest, where ecologically and taxonomically similar mosquito species coexist. These similar species usually exploit limiting resources in different ways, such as feeding at different heights inside the bromeliad water column at micro-habitat scale, colonizing either terrestrial or epiphytic bromeliads at the habitat scale and selecting more or less physically stressful habitats at landscape scale. Thus, resource partitioning may be an important structuring process in these assemblages. Mosquito species diversity was at least partly caused by the several ways in which potential competitors have evolved specialized traits.

*Anopheles bellator* and *Cx. imitator* significantly co-occurred in the same bromeliad, especially on scrublands. Both species are frequent in *Quesnelia* bromeliads that are highly exposed to sunlight. In these bromeliads, larvae of both species collect and filter detritus and microorganisms [16]. Because of the lack of a respiratory siphon which characterizes all *Anopheles* species, *An. bellator* feed at the air-water interface, whilst *Cx. imitator* feeds mainly in the water column, as do other *Culex* species [36]. Similarly, Marques et al. [31] observed statistically significant associations between other species of *Anopheles* (*Kerteszia*) and *Culex* (*Microculex*) in *Vriesea* and *Nidularium* bromeliads in lowlands and hillsides at another study site in the Atlantic rainforest. The non-random co-occurrence of these species at micro-habitat scale is indicative of the first mechanism of species coexistence in mosquito assemblages (Figure 1A). Prior to Gilbert et al. [10], Yee et al. [20] proposed this mechanism after video recording the feeding behavior of *Cx. pipiens*, *Ae. albopictus* and *Ae. triseriatus* specimens in different food environments within an experimental study design. Interestingly, this is a consistent explanation for the co-occurrence of *An. bellator* with *Cx. imitator* in the present work and *An. cruzii* with other *Cx*. (*Microculex*) species and *An. homunculus* with *Cx. ocellatus* in Marques et al. [31].

Mosquito species richness varied non-linearly across the gradient of vegetation gradient. More species occurred in forested habitats than on scrublands, showing that only some mosquito species can endure the physiological stress of harsh environments. More species occurred in the forest than was expected, attributable to diverse phytotelmata-inhabiting mosquito species of the tribe Sabethini [37]. However, the maximum species richness was observed in the middle where mixed vegetation occurs, with both arboreal and scrub elements. This represented a transition zone (i.e., an ecotone, between scrubland and dense ombrophilous forest). As expected, this is part of the gradient with highest heterogeneity of vegetation complexity. The resulting mosaic of trees and bushes caused variation in light incidence and thus the occurrence of both terrestrial and epiphytic bromeliads. *Wyeomyia* (*Phoniomyia*) mosquito species occurred in this physiognomy. The feeding behavior of these species should be very similar at the micro-habitat scale and because of that these species did not co-occur in the same type of bromeliad. *Wyeomyia muhelensis* occurred in terrestrial *Quesnelia* bromeliads, whereas *Wy. quasilongirostris* occurred in epiphytic *Vriesea* bromeliads. Hence, coexistence probably is possible because of the presence of these terrestrial and epiphytic bromeliad species and provides evidence in support of the second mechanism of species coexistence in mosquito assemblages (Figure 1B).

The pairs of potential competitor species *Anopheles bellator*-*An. cruzii* and *Ae. scapularis*-*Ae. serratus* did not co-occur at micro-habitat and habitat. However, all species did co-occur at the landscape scale. Because both pairs of species are taxonomically and ecologically similar, they cannot possibly allow their persistence at local scales which may suggest a mechanism of coexistence at the landscape scale. This observational evidence may be related to the asymmetrical competition mechanism which results in a greater likelihood of local competitive exclusion, but it can provide a regional coexistence depending on the context [12]. This finding represents evidence for the third mechanism of species coexistence in mosquito assemblages (Figure 1C).

## Conclusions

The present work did not experimentally test the effects of competition among species across the gradient of vegetation physiognomies to ensure that the observed patterns match the predicted mechanism. Thus, it was found herein empirical evidence for three mechanisms of resource-niche partitioning that provided greater biodiversity in the species-rich mosquito community of Atlantic rainforest. The mechanisms are: 1) spatial niche segregation in vertical strata in bromeliads, 2) niche partitioning mediated by spatial heterogeneity which promotes species coexistence at habitat scale and 3) asymmetrical competition with context dependence in groups of taxonomically similar species in bromeliads and ephemeral pools.

## Additional files

Additional file 1: Table S1 Distribution of the foliage vertical length (in meters) per forest strata, transect, and collection site, Parque Estadual da Ilha do Cardoso, Cananéia, São Paulo State, Brazil, 2009. Additional file 2: Table S2 Results of the AIC Model Selection approach with mosquito (adult females) individual abundance in function of vegetation gradient, Parque Estadual da Ilha do Cardoso, 2009-2010. Additional file 3: Table S3 Distribution of the number of collected adult mosquitoes in CDC traps (Light and CO_2_) per species and collection site, Parque Estadual da Ilha do Cardoso, Cananéia, São Paulo State, Brazil, 2009-2010. Additional file 4: Figure S1-S4 The abundance of a given species as adults per collection site as a function of vegetation gradient. Additional file 5: Table S4 Distribution of numbers of larvae of mosquito species per bromeliad in a gradient of vegetation, Parque Estadual da Ilha do Cardoso, southeastern Atlantic Forest, Brazil, 2009-2010. Additional file 6: Table S5 Results of the AIC Model Selection approach with mosquito species richness in function of vegetation gradient and its heterogeneity, Parque Estadual da Ilha do Cardoso, 2009-2010. Additional file 7: Figure S5 Solid lines represent GAM fitted to the data using cubic regression splines. Dashed lines define the 95% CI. A) Figure 4B-*Wy. muhelensi*. B) Figure 4B-*Wy. quasilongirostris*. C) Figure 4D-*Ae. scapularis*. D) Figure 4D-*Ae. serratus*. Additional file 8: Figure S6 Solid lines represent GAM fitted to the data using cubic regression splines. Dashed lines define the 95% CI. A) Figure 5A. B) Figure 5B. Additional file 9: Figure S7 Solid lines represent GAM fitted to the data using cubic regression splines. Dashed lines define the 95% CI. A) Figure 4A-*An. bellator*. B) Figure 4A-*Cx. imitator*. C) Figure 4C-*An. bellator*. D) Figure 4C-*An. cruzii*.

## Abbreviations

AIC: Akaike Information Criteria
CDC: Centers for Disease Control
CI: Confidence Interval
GAM: Generalized Additive Models
HOF: Huisman-Olff-Fresco
PCA: Principal Component Analysis
PEIC: Parque Estadual da Ilha do Cardoso
OR: Odds Ratio
