# Supplementary material for: Coexistence mechanisms at multiple scales in mosquito assemblages
Source: BMC Ecol. 2014 Nov 11;14:30. doi: 10.1186/s12898-014-0030-8 (PMC4247778; doi:10.1186/s12898-014-0030-8)
Supplement: Additional file 1: Table S1 — Distribution of the foliage vertical length (in meters) per forest strata, transect, and collection site, Parque Estadual da Ilha do Cardoso, Cananéia, São Paulo State, Brazil, 2009. [file 12898_2014_30_MOESM1_ESM.pdf]

Table S1. Distribution of the foliage vertical length (in meters) per forest strata, transect, and collection site, Parque Estadual da Ilha do Cardoso, Cananéia, São Paulo State, Brazil, 2009.

		Forest Strata				
**Collection site**	**Transect**	**0 - 1 m**	**1 - 5 m**	**5 - 10 m**	**10 - 15 m**	**> 15 m**
P1	South	0	0.6	0.6	0	0
	East	0	0.2	0.5	0	0
	North	0.1	1.8	1	0	0
	West	0.1	0.1	0.8	0	0
P2	South	0.1	0.1	1	0	0
	East	0.1	0.1	0.6	0	0
	North	0.2	0.2	1.2	0	0
	West	0.3	0	0	0	0
P3	South	0.9	0.2	1.4	0.8	0
	East	0.3	1.2	0.5	0.6	0
	North	0.4	0	0.8	0.7	0
	West	0.9	1.6	0	0.6	0
P4	South	0.5	0.3	0.1	0	0
	East	0.8	0.3	0.1	1.1	0
	North	0.3	0.8	0.6	0	0
	West	0.3	1.3	1.5	0	0
P5	South	0.8	0.3	3.6	1.1	0
	East	0.4	0	0.9	2.9	0
	North	0.2	1.4	0.5	1.5	0
	West	0.9	1.4	0.9	1.5	0
P6	South	0.7	0.9	2.6	1.5	0
	East	0.5	0	0	0.5	0
	North	0.7	0.5	0.4	0	0
	West	0.6	0.3	0.2	1	0
P7	South	0.7	0.2	0.1	1.1	0
	East	0.5	1.1	0.7	0	0
	North	0.5	0	0.1	0	0
	West	0.9	1	0.3	0.4	0
P8	South	0.9	1.5	0	0	0
	East	0.9	2.3	0	0	0
	North	0.5	1	0	0	0
	West	0.9	1.4	0.6	0	0
P9	South	0.1	0.4	0.2	0.5	0
	East	0.2	2.2	1.7	0.6	0
	North	0.7	1.8	2.6	1.9	0
	West	0.3	0.5	0.5	0	0
P10	South	0.2	0.1	1.8	1.5	0
	East	0.2	0.1	0.1	1.5	0
	North	0.1	0	1	0.5	0
	West	0.2	0.2	0	0.1	0
P11	South	0.1	1	0.4	0	0

Table A1. *Continuation.*

	East	0.1	0.5	1.4	0	0
	North	0.1	0.9	0.5	0	0
	West	0	0.1	1.3	0	0
P12	South	0.6	0.6	0.2	0	1
	East	0	0	0	1.5	0
	North	0.1	0	3	2	0
	West	0	0.8	1.5	1	0
P13	South	0	0.1	0	1	1.5
	East	0	1.6	2.5	0.5	0
	North	0	1	1	1.5	2
	West	0	1	2.5	4.3	2.9
P14	South	0	0	0.7	0.1	2.9
	East	0	0	1.1	0	3.5
	North	0	0.5	0.5	0.2	2.4
	West	0	0	1.1	0	1.5
P15	South	0	0	0.2	0.7	3.7
	East	0	0.3	0.5	0.3	2.9
	North	0	0.1	0	1	2.5
	West	0	0	0	2.8	1
P16	South	0	0.1	0.2	0	1.1
	East	0	0.7	0.6	0.1	3.4
	North	0	0.4	0.4	1.2	1.5
	West	0	0.1	0.2	0.1	1.5
P17	South	0	0.9	4	0.2	0
	East	0	0.4	0.1	1.1	2
	North	0	0.4	0.6	1.3	2.5
	West	0	1.5	0.9	2.6	0
P18	South	0	0	0.3	1.6	5.6
	East	0	0.2	0.7	0.4	1.5
	North	0	0.1	0.7	1	0.9
	West	0.1	1.2	0.8	1	2.7
P19	South	0	0.2	0.4	0.6	0.7
	East	0.2	1.1	0.2	0.3	0.7
	North	0	0.2	2.8	1.5	2
	West	0	0.7	2.2	0.5	0
P20	South	0	0.1	2.2	0.2	3
	East	0	0.1	0.1	1	2
	North	0	0	0.1	0.2	2.5
	West	0	0	0.4	0	2
P21	South	1	1.2	0	0	0
	East	0.3	1.2	0	0	0
	North	0.9	2	0	0	0
	West	0.2	0.4	0	0	0
P22	South	0.5	0	0	0	0

Table A1. *Continuation.*

	East	0.6	0	0	0	0
	North	1	0.9	0	0	0
	West	0.7	0	0	0	0
P23	South	1	0.5	0	0	0
	East	0.5	2.1	0	0	0
	North	0.9	2.3	0	0	0
	West	0.3	0.6	0	0	0
P24	South	0.9	0.2	0	0	0
	East	0.6	1.6	0	0	0
	North	0.4	2.6	0.6	0	0
	West	1	0.5	0	0	0
P25	South	0.6	1	0	0	0
	East	0.7	1.6	0	0	0
	North	0.3	0.9	0.3	0	0
	West	0.9	2.9	0	0	0
P26	South	0.2	1.6	0	0	0
	East	0.4	1.8	0	0	0
	North	0.9	0.9	0	0	0
	West	0.9	2.1	0	0	0
P27	South	0.9	1.2	0	0	0
	East	0.6	1.8	0	0	0
	North	0.9	2.6	0	0	0
	West	0.9	1.1	0	0	0
P28	South	0.8	0	0	0	0
	East	0.5	0	0	0	0
	North	0.4	0.4	0	0	0
	West	0.1	0	0	0	0
P29	South	0.4	0	0	0	0
	East	0.4	0	0	0	0
	North	0.4	1.5	0	0	0
	West	0.9	1.6	0	0	0
P30	South	0.9	1.2	0	0	0
	East	0.4	1.2	0	0	0
	North	0.8	1.3	0	0	0
	West	0.7	0.5	0	0	0
