# Supplementary material for: Coexistence mechanisms at multiple scales in mosquito assemblages
Source: BMC Ecol. 2014 Nov 11;14:30. doi: 10.1186/s12898-014-0030-8 (PMC4247778; doi:10.1186/s12898-014-0030-8)
Supplement: Additional file 2: Table S2 — Results of the AIC Model Selection approach with mosquito (adult females) individual abundance in function of vegetation gradient, Parque Estadual da Ilha do Cardoso, 2009-2010. [file 12898_2014_30_MOESM2_ESM.pdf]

Table S2. Results of the AIC Model Selection approach with mosquito (adult females) individual abundance in function of vegetation gradient, Parque Estadual da Ilha do Cardoso, 2009-2010.

Species	Models	df[a]	AICc	AICc Weight
***An. bellator* individual abundance** *in function of* **vegetation gradient** (Fig. 4A, 4C)	Model I (flat)	1	75.28	0.00
	Model II monotone)	2	**64.53**[b]	**0.54**
	Model III (plateau)	3	67.01	0.16
	Model IV (symmetric)	3	67.01	0.16
	Model V (skewed)	4	69.69	0.04
	Model VI (bimodal symmetric)	4	68.25	0.08
	Model VII (bimodal skewed)	5	71.14	0.02
***Cx. imitator* individual abundance** *in function of* **vegetation gradient** (Fig. 4A)	Model I (flat)	1	561.51	0.00
	Model II monotone)	2	**402.54**	**0.57**
	Model III (plateau)	3	405.02	0.17
	Model IV (symmetric)	3	405.02	0.17
	Model V (skewed)	4	407.69	0.04
	Model VI (bimodal symmetric)	4	407.69	0.04
	Model VII (bimodal skewed)	5	410.59	0.01
***Wy. muhelensi* individual abundance** *in function of* **vegetation gradient** (Fig. 4B)	Model I (flat)	1	3196	0.00
	Model II (monotone)	2	3140	0.00
	Model III (plateau)	3	2937	0.00
	Model IV (symmetric)	3	2097	0.00
	Model V (skewed)	4	**1856**	**1.0**
	Model VI (bimodal symmetric)	4	2100	0.00
	Model VII (bimodal skewed)	5	2001	0.00
***Wy. quasilongirostris* individual abundance** *in function of* **vegetation gradient** (Fig. 4B)	Model I (flat)	1	4529.34	0.00
	Model II (monotone)	2	4387.42	0.00
	Model III (plateau)	3	4078.91	0.00
	Model IV (symmetric)	3	2652.23	0.09
	Model V (skewed)	4	**2647.77**	**0.88**
	Model VI (bimodal symmetric)	4	2654.91	0.02
	Model VII (bimodal skewed)	5	2657.81	0.01
***An. cruzii* individual abundance** *in function of* **vegetation gradient** (Fig. 4C)	Model I (flat)	1	3022	0.00
	Model II (monotone)	2	**2016**	**0.55**
	Model III (plateau)	3	2018	0.20
	Model IV (symmetric)	3	2018	0.20
	Model V (skewed)	4	2021	0.05
	Model VI (bimodal symmetric)	4	-	-
	Model VII (bimodal skewed)	5	-	-
***Ae. scapularis* individual abundance** *in function of* **vegetation gradient** (Fig. 4D)	Model I (flat)	1	68584.01	0.0
	Model II (monotone)	2	55052.87	0.0
	Model III (plateau)	3	50665.98	0.0
	Model IV (symmetric)	3	37526.66	0.17
	Model V (skewed)	4	**37523.98**	**0.63**
	Model VI (bimodal symmetric)	4	37526.65	0.17
	Model VII (bimodal skewed)	5	37529.56	0.03
***Ae. serratus* individual abundance** *in function of* **vegetation gradient** (Fig. 4D)	Model I (flat)	1	2152.84	0.0
	Model II (monotone)	2	1894.79	0.0
	Model III (plateau)	3	1699.88	0.0
	Model IV (symmetric)	3	1760.84	0.0
	Model V (skewed)	4	1692.37	0.0
	Model VI (bimodal symmetric)	4	**1654.32**	**0.73**
	Model VII (bimodal skewed)	5	1656.93	0.27

[a]: The number of parameters in the Model Selection varies, accordingly: flat, one parameter; monotone, two parameters; plateau, three parameters; symmetric, three parameters; skewed, four parameters; bimodal symmetric, four parameters and bimodal skewed, five parameters.

[b]: The results printed in bold represent the most plausible model under the lowest value of Akaike Information Criteria based on the best model's parsimony and likelihood [34].
