# Supplementary material for: Coexistence mechanisms at multiple scales in mosquito assemblages
Source: BMC Ecol. 2014 Nov 11;14:30. doi: 10.1186/s12898-014-0030-8 (PMC4247778; doi:10.1186/s12898-014-0030-8)
Supplement: Additional file 3: Table S3 — Distribution of the number of collected adult mosquitoes in CDC traps (Light and CO2) per species and collection site, Parque Estadual da Ilha do Cardoso, Cananéia, São Paulo State, Brazil, 2009-2010. [file 12898_2014_30_MOESM3_ESM.pdf]

Table S3. Distribution of the number of collected adult mosquitoes in CDC traps (Light and CO_2) per species and collection site, Parque Estadual da Ilha do Cardoso, Cananéia, São Paulo State, Brazil, 2009-2010.

Species	P01[a]	P02	P03	P04	P05	P06	P07	P08	P09	P10	P11	P12	P13	P14	P15	P16	P17	P18	P19	P20	P21	P22	P23	P24	P25	P26	P27	P28	P29	P30
Aedes albifasciatus	0	0	0	0	0	0	0	0	0	0	0	0	0	0	0	0	0	0	0	0	4	5	1	0	1	0	2	0	0	0
Aedes fulvus	1	0	1	0	0	0	0	0	2	1	0	0	1	0	0	0	0	0	0	0	0	0	0	0	0	0	0	0	0	0
Aedes hastatus	7	0	0	0	0	0	0	0	0	0	1	0	2	6	5	0	0	1	1	0	0	0	1	1	0	1	0	0	0	0
Aedes oligopistus	2	1	0	0	1	0	0	0	0	1	4	2	3	5	7	0	1	2	0	0	0	0	0	0	0	0	0	1	3	1
Aedes perventor	1	1	17	2	7	2	2	0	3	1	1	0	0	0	0	0	1	0	0	0	0	0	0	0	0	1	0	0	0	0
Aedes scapularis	47	4	6	0	5	2	6	0	1	2	3	0	0	2	1	2	2	1	3	0	257	16	39	16	64	53	5	4	32	59
Aedes serratus	30	9	4	3	8	9	0	0	3	18	17	21	14	26	20	9	17	0	5	1	5	0	0	0	13	5	1	0	4	1
Anopheles bellator	1	1	0	1	1	3	1	0	5	0	1	2	1	1	1	0	0	0	0	0	10	1	2	4	0	6	2	0	0	1
Anopheles cruzii	2	2	8	3	6	24	1	2	32	15	3	9	10	12	18	5	7	37	27	22	1	1	1	0	5	11	1	1	1	1
Anopheles homunculus	2	0	1	0	0	0	0	0	0	0	0	0	5	0	1	2	3	6	6	0	0	0	0	0	0	1	0	0	0	0
Anopheles maculipes	0	0	0	0	0	0	0	0	0	0	0	0	0	0	0	0	0	0	0	0	4	4	6	6	2	7	1	3	1	1
Anopheles mediopunctatus	2	1	0	0	1	0	1	0	6	0	0	2	0	1	0	0	1	0	0	0	0	0	0	0	1	0	0	0	0	0
Coquillettidia chrysonotum	404	137	267	355	209	510	325	438	2333	186	375	83	116	90	106	53	94	85	40	87	108	73	116	141	456	445	210	82	72	85
Coquillettidia venezuelensis	0	0	1	0	0	1	1	0	0	2	1	3	3	0	0	0	0	1	0	0	0	0	0	0	0	1	0	0	0	2
Culex abonnenci	1	1	0	0	0	0	0	0	0	0	0	0	0	0	0	0	0	0	0	0	0	0	0	0	0	0	0	0	0	0
Culex akritos	0	5	0	0	0	2	7	35	16	17	34	12	5	12	1	1	0	0	0	0	0	0	0	0	0	1	0	0	0	0
Culex aliciae	0	0	0	0	0	0	0	0	0	0	0	0	1	0	0	0	0	0	0	0	0	0	0	0	0	0	0	0	0	0
Culex alinkios	0	0	0	0	0	0	0	0	0	0	0	0	1	0	0	0	0	0	0	0	0	0	0	0	0	0	0	0	0	0
Culex aphylactus	0	0	1	2	0	0	0	0	0	0	0	0	0	0	0	0	0	0	0	0	0	0	0	1	0	0	0	0	0	0
Culex aureonotatus	0	0	0	0	0	0	0	0	0	0	0	0	0	0	0	0	0	0	0	0	0	0	1	0	0	0	0	0	0	0
Culex bidens	0	0	0	1	0	0	1	0	0	0	0	0	0	0	0	0	0	0	0	1	0	0	0	0	0	0	0	0	1	0
Culex declarator	1	0	0	0	0	2	3	7	5	2	3	2	0	0	0	0	0	0	0	0	0	0	0	0	0	0	0	1	0	0
Culex dureti	8	13	1	1	0	0	0	0	1	2	1	0	1	0	0	0	0	0	0	0	0	0	0	0	0	0	0	0	0	0
Culex faurani	3	0	0	0	1	0	5	0	1	0	0	1	0	0	0	0	0	0	0	0	0	0	0	0	0	0	0	0	0	0
Culex gairus	0	0	0	0	0	0	0	0	0	0	0	0	0	0	0	0	0	0	0	0	0	0	0	0	0	0	1	0	0	1

Table S3. *Continuation.*

Culex galvaoi	2	0	2	0	0	0	0	0	0	0	0	0	0	0	0	0	0	0	0	0	0	0	0	0	0	0	0	0	0	0
Culex imitator	8	8	3	1	2	0	0	1	0	0	0	0	0	0	0	0	0	1	0	0	1	1	1	4	1	19	12	0	2	8
Culex inadimirabilis	0	0	0	0	0	0	0	0	0	0	0	0	0	0	0	0	0	0	0	0	20	20	43	30	14	53	5	3	18	4
Culex inimitabilis fuscatus	1	0	0	0	0	0	0	0	0	0	0	0	0	0	0	0	0	0	0	0	0	0	0	0	0	0	0	0	0	0
Culex intrincatus	0	0	0	0	0	0	0	0	0	0	0	0	0	0	0	0	0	0	0	0	0	0	0	1	0	0	0	0	0	0
Culex microphyllus	0	0	0	0	0	0	0	0	0	0	0	0	0	0	0	0	0	1	0	0	0	0	0	1	0	1	0	0	0	0
Culex misionensis	0	2	1	1	0	0	0	0	0	0	0	0	0	0	0	0	0	0	0	0	0	0	0	0	0	0	0	0	0	0
Culex mollis	0	0	0	0	0	1	0	0	0	0	0	0	0	0	0	0	0	0	0	0	0	0	0	0	0	0	0	0	0	0
Culex neglectus	1	0	3	3	1	0	0	1	0	0	0	2	0	0	0	1	0	0	0	0	0	0	0	1	0	1	0	0	0	2
Culex ocellatus	0	0	0	0	0	0	0	0	0	0	0	0	0	0	0	0	0	0	3	0	0	0	0	0	0	0	0	0	0	0
Culex ocossa	0	0	0	0	0	0	0	0	0	0	0	0	0	0	0	0	0	0	0	0	0	0	0	0	0	2	1	0	0	0
Culex oedipus	0	0	0	0	0	0	0	0	0	0	0	0	0	0	0	0	0	0	0	0	0	0	0	0	0	1	0	0	0	0
Culex pedroi	2	3	1	0	0	0	3	2	10	0	4	0	0	0	0	1	0	0	0	0	0	0	0	0	0	0	0	0	0	0
Culex pilosus	0	0	0	0	0	0	0	0	0	0	0	0	0	0	0	0	0	0	0	0	0	0	0	0	0	0	1	0	0	0
Culex putumayensis	6	3	6	1	3	0	0	0	0	0	0	5	0	0	0	0	0	0	0	0	0	0	0	0	0	0	0	0	0	0
Culex rabelloi	2	3	0	0	0	0	0	0	0	0	0	3	0	0	0	0	0	0	0	0	0	0	0	0	0	0	0	0	0	0
Culex ribeirensis	5	8	1	1	1	0	6	1	0	0	5	2	0	0	1	1	0	0	0	0	5	2	9	12	27	58	0	1	0	0
Culex sachettae	75	74	10	7	5	19	71	89	66	16	70	4	10	12	22	25	3	1	0	0	2	3	2	4	3	3	0	1	0	0
Culex vaxus	0	0	0	0	0	0	0	0	0	0	0	0	0	0	0	0	0	0	0	0	0	0	0	1	0	0	0	0	0	0
Limatus durhami	0	0	0	0	0	0	0	0	1	2	1	0	6	7	16	9	8	3	0	3	0	0	0	0	0	0	0	0	0	0
Limatus flavisetosus	0	0	0	0	0	0	0	0	0	1	2	0	0	3	6	4	1	2	0	0	0	0	0	0	0	0	0	0	0	0
Mansonia pseudotitillans	0	0	1	0	0	0	0	0	0	0	0	0	0	0	0	0	0	0	0	0	0	0	0	0	0	0	0	0	0	0
Psorophora albigenu	2	2	2	3	2	1	0	0	0	0	0	0	0	0	0	0	0	0	0	0	0	0	0	0	0	0	0	0	0	0
Psorophora albipes	0	0	3	1	4	0	0	0	0	0	0	0	0	0	0	0	0	0	0	0	0	0	0	0	0	0	0	0	0	0
Psorophora ferox	20	16	1	9	2	4	1	0	3	6	2	2	1	0	4	0	0	0	0	0	1	0	3	1	1	1	0	0	0	0
Psorophora saeva	0	0	0	0	0	0	0	0	0	0	0	0	0	0	0	0	0	0	0	0	0	0	1	0	0	0	0	0	0	0

Table S3. *Continuation*.

Runchomyia cerqueirai	0	0	0	0	0	0	0	0	0	3	1	0	0	1	0	0	0	0	0	0	0	0	0	0	0	0	0	0	0	0
Runchomyia frontosa	0	0	0	0	0	0	0	0	0	0	0	0	0	0	0	0	0	2	1	0	0	0	0	0	0	0	0	0	0	0
Runchomyia humboldti	0	0	0	0	0	0	0	0	1	3	0	0	0	0	0	0	1	0	0	0	0	0	0	0	0	0	0	0	0	0
Runchomyia reversa	12	1	7	2	3	1	1	3	3	16	2	0	1	0	2	1	1	0	0	0	0	0	0	0	0	0	0	0	0	0
Runchomyia theobaldi	1	0	0	2	0	1	0	1	3	8	2	0	1	1	6	3	1	1	0	0	0	0	0	0	0	0	0	0	0	0
Sabethes intermedius	0	0	0	0	0	0	0	0	0	0	0	0	0	0	0	1	0	0	0	0	0	0	0	0	0	0	0	0	0	0
Sabethes soperi	0	0	0	0	0	0	0	0	1	0	0	0	0	0	0	0	0	0	0	0	0	0	0	0	0	0	0	0	0	0
Trichoprosopon pallidiventer	0	0	0	0	0	0	0	0	0	1	0	0	1	0	0	0	0	0	1	1	0	0	0	0	0	0	0	0	0	0
Uranotaenia calosomata	0	0	0	0	0	0	0	0	0	0	0	0	1	1	0	0	0	0	0	0	0	0	0	0	0	0	0	0	0	0
Uranotaenia geometrica	1	0	0	0	0	0	0	0	0	0	0	0	0	0	0	0	0	0	0	0	0	0	0	0	0	0	0	0	0	1
Uranotaenia incognita	5	0	1	1	1	2	1	0	2	0	1	1	0	0	0	0	0	0	0	0	0	0	0	0	0	0	0	0	0	0
Uranotaenia mathesoni	0	0	0	0	0	0	0	0	0	0	0	0	0	0	0	1	0	0	0	0	0	0	0	0	0	0	0	0	0	0
Uranotaenia pallidoventer	0	0	1	0	0	0	0	0	0	0	0	1	0	0	0	0	0	1	0	0	0	0	0	0	0	0	0	0	0	0
Wyeomyia aporonoma	0	0	0	0	0	0	0	0	1	0	0	2	0	0	0	1	1	2	0	0	0	0	0	0	0	0	0	0	0	0
Wyeomyia confusa	0	0	0	0	0	0	0	0	1	0	0	2	0	3	4	1	4	0	0	0	0	0	0	0	0	0	0	0	0	0
Wyeomyia mulhensi	1	15	5	0	1	8	17	20	50	20	0	0	0	0	0	0	0	6	2	0	0	0	5	5	0	0	0	0	0	0
Wyeomyia occulta	0	0	0	0	0	0	0	0	1	0	0	0	0	0	0	0	0	0	0	0	0	0	0	0	0	0	0	0	0	0
Wyeomyia pallidoventer	4	4	1	1	0	1	3	0	24	12	10	0	1	0	0	0	0	0	0	0	0	0	0	0	0	0	0	0	0	0
Wyeomyia pertinens	1	1	0	0	0	0	0	1	0	0	1	0	0	0	0	1	1	0	0	0	0	0	0	0	0	0	0	0	0	0
Wyeomyia quasilongirostris	15	36	27	8	4	8	42	20	40	17	5	1	2	0	0	3	0	1	13	4	0	0	5	7	0	0	0	0	0	0
Wyeomyia shannoni	0	0	0	0	0	0	0	0	0	0	1	0	0	0	0	0	0	0	0	0	0	0	0	0	0	0	0	0	0	0
Wyeomyia theobaldi	0	3	3	1	0	1	2	1	3	2	1	0	0	0	0	0	0	0	3	0	0	0	0	0	0	0	0	0	0	0

[a]: Collection points P1-P10 are from mixed arboreal and scrub vegetation, P11-P20 are from dense ombrophilous forest and P21-P30 are from scrub vegetation.
