# Supplementary material for: Coexistence mechanisms at multiple scales in mosquito assemblages
Source: BMC Ecol. 2014 Nov 11;14:30. doi: 10.1186/s12898-014-0030-8 (PMC4247778; doi:10.1186/s12898-014-0030-8)
Supplement: Additional file 4: Figure S1-S4 — The abundance of a given species as adults per collection site as a function of vegetation gradient. [file 12898_2014_30_MOESM4_ESM.pdf]

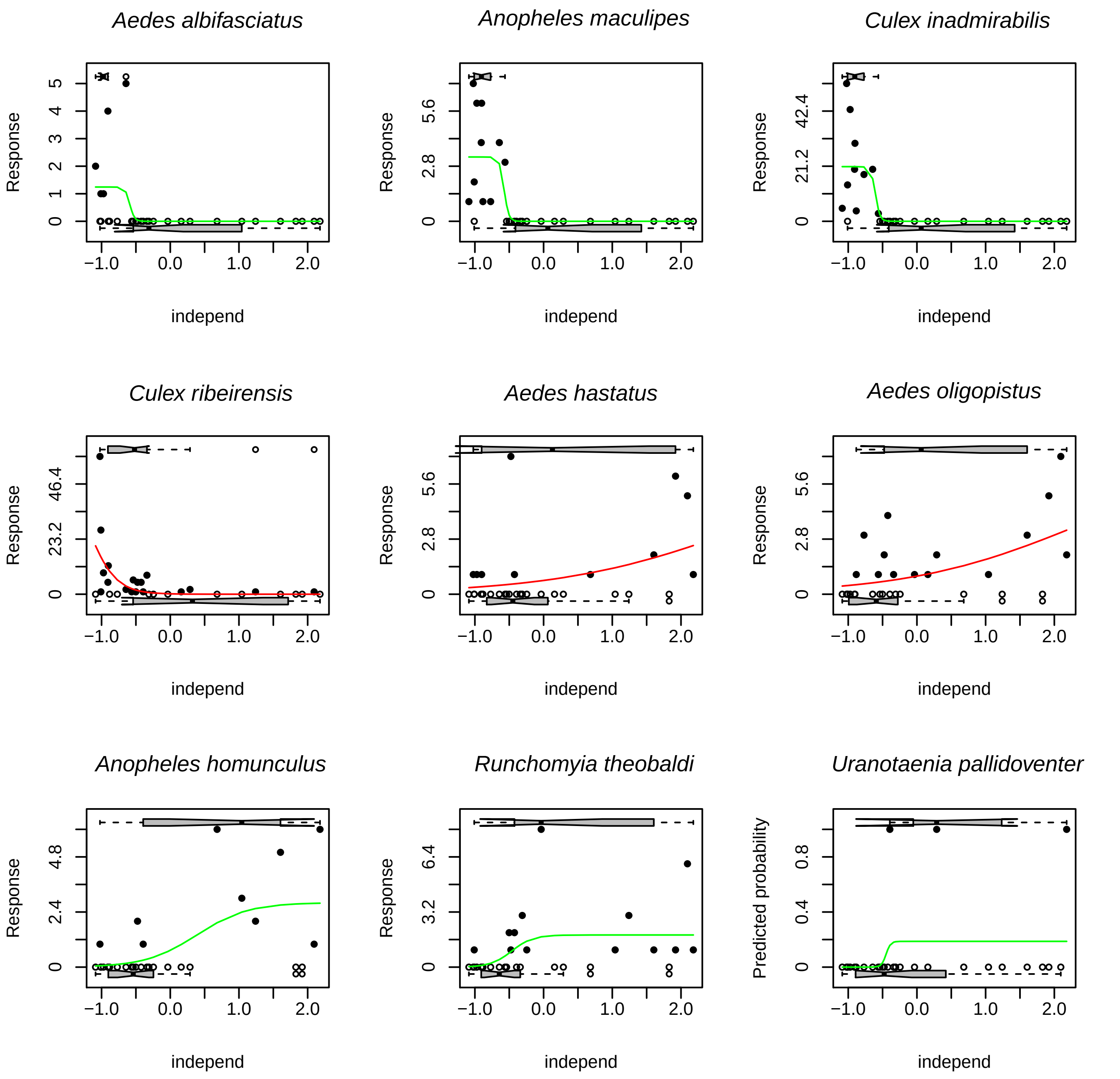
Aedes albifasciatus
Anopheles maculipes
Culex inadmirabilis
Culex ribeirensis
Aedes hastatus
Aedes oligopistus
Anopheles homunculus
Runchomyia theobaldi
Uranotaenia pallidoventer
Response
Predicted probability
independ
−1.0
0.0
1.0
2.0
0
1
2
3
4
5
2.8
5.6
21.2
42.4
23.2
46.4
2.4
4.8
3.2
6.4
0.4
0.8

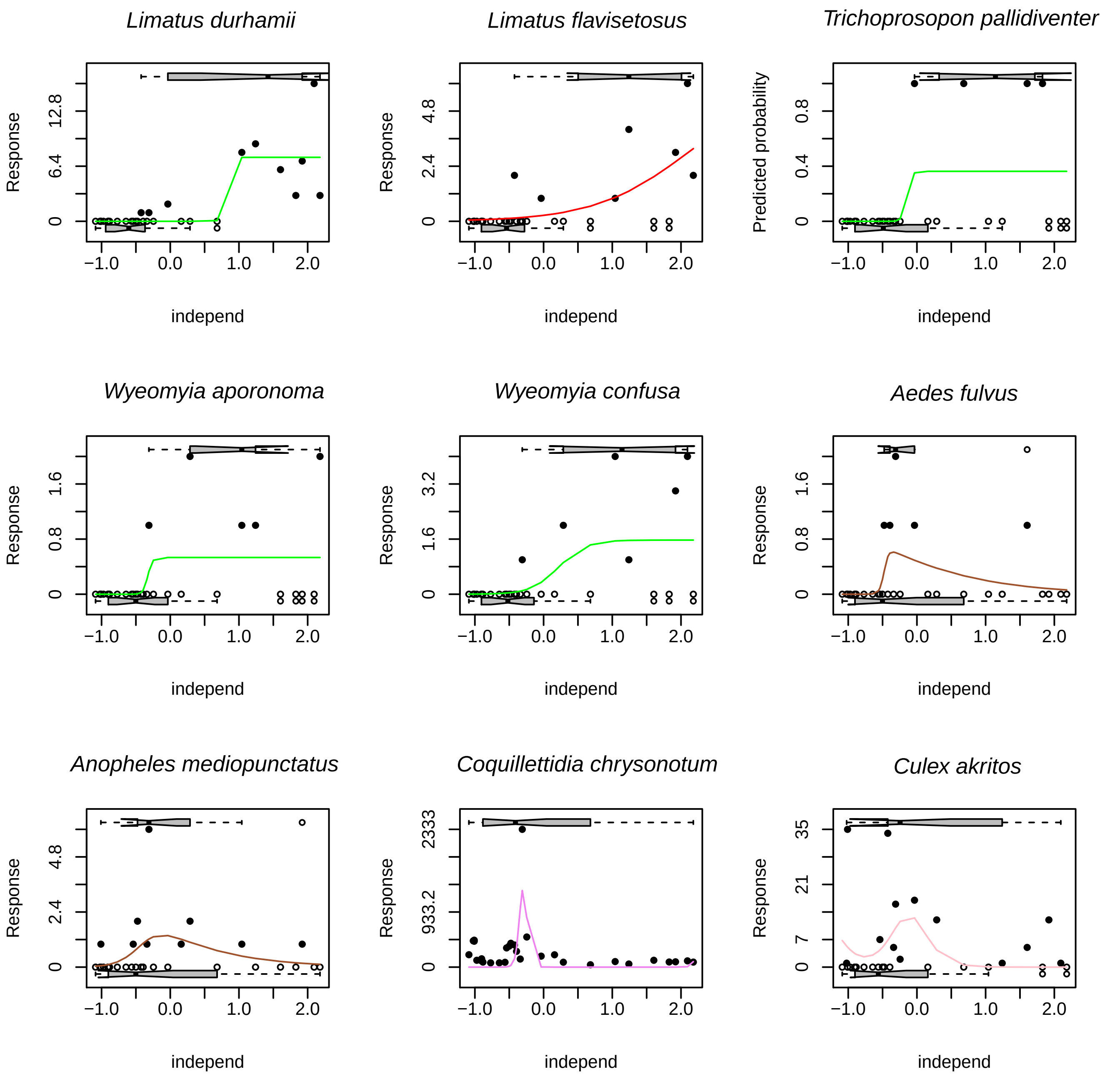

Limatus durhamii
Limatus flavisetosus
Trichoprosopon pallidiventer
Wyeomyia aporonoma
Wyeomyia confusa
Aedes fulvus
Anopheles mediopunctatus
Coquillettidia chrysonotum
Culex akritos
Response
Predicted probability
independ
−1.0
0.0
1.0
2.0
0
6.4
12.8
2.4
4.8
0.4
0.8
1.6
3.2
933.2
2333
7
21
35

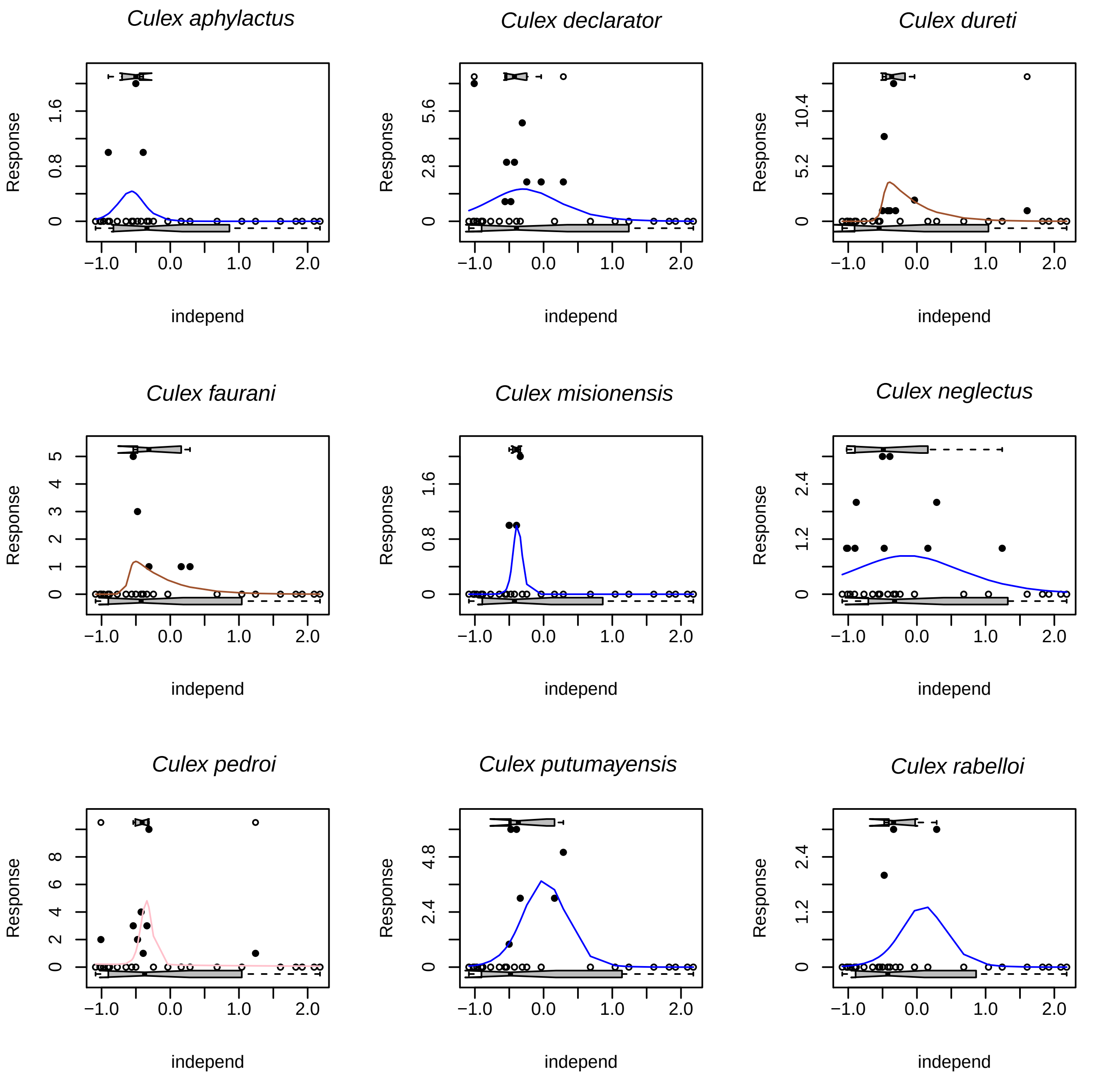
Culex aphylactus
Culex declarator
Culex dureti
Culex faurani
Culex misionensis
Culex neglectus
Culex pedroi
Culex putumayensis
Culex rabelloi
Response
independ
−1.0
0.0
1.0
2.0
0
0.8
1.6
2.8
5.6
5.2
10.4
1
2
3
4
5
1.2
2.4
6
8
4.8

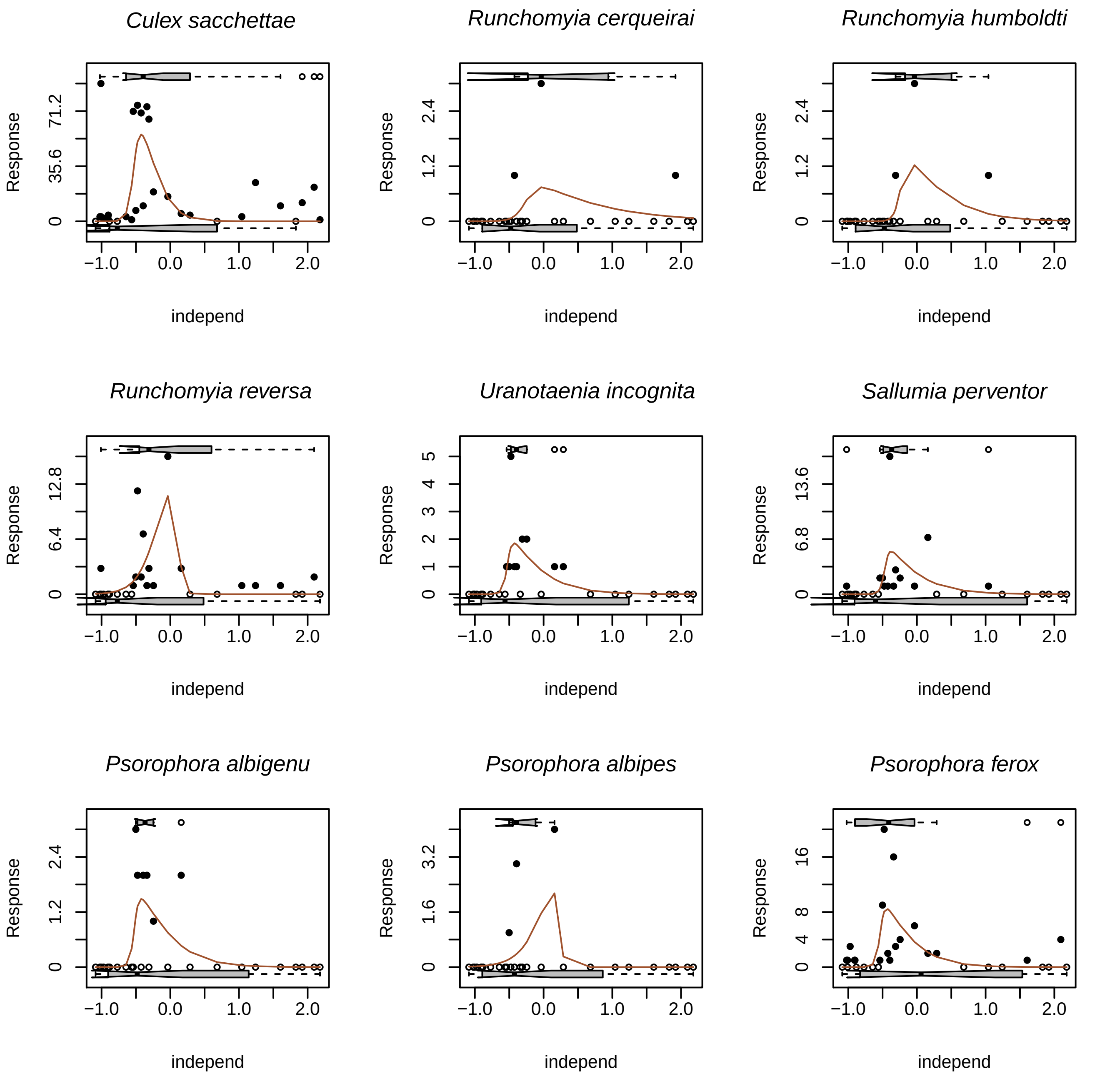

Culex sacchettae
Runchomyia cerqueirai
Runchomyia humboldti
Runchomyia reversa
Uranotaenia incognita
Sallumia perventor
Psorophora albigenu
Psorophora albipes
Psorophora ferox
Response
independ
