# Supplementary material for: Coexistence mechanisms at multiple scales in mosquito assemblages
Source: BMC Ecol. 2014 Nov 11;14:30. doi: 10.1186/s12898-014-0030-8 (PMC4247778; doi:10.1186/s12898-014-0030-8)
Supplement: Additional file 5: Table S4 — Distribution of numbers of larvae of mosquito species per bromeliad in a gradient of vegetation, Parque Estadual da Ilha do Cardoso, southeastern Atlantic Forest, Brazil, 2009-2010. [file 12898_2014_30_MOESM5_ESM.pdf]

**Table S4**. Distribution of numbers of larvae of mosquito species per bromeliad in a gradient of vegetation, Parque Estadual da Ilha do Cardoso, southeastern Atlantic Forest, Brazil, 2009-2010.

| Bromeliad number[a]: | *An. cruzii* | *An. bellator* | *Cx. imitator* | *Wy. quasilongirostris* | *Wy. muhelensis* |
|---|---|---|---|---|---|
| 1 | 2 | 1 | 1 | 1 | 0 |
| 2 | 0 | 1 | 0 | 0 | 1 |
| 3 | 9 | 1 | 1 | 0 | 1 |
| 4 | 2 | 0 | 0 | 0 | 1 |
| 5 | 0 | 2 | 0 | 0 | 1 |
| 6 | 4 | 1 | 1 | 1 | 0 |
| 7 | 2 | 0 | 1 | 0 | 1 |
| 8 | 0 | 1 | 1 | 1 | 0 |
| 9 | 0 | 0 | 0 | 1 | 0 |
| 10 | 2 | 0 | 0 | 1 | 0 |
| 11 | 0 | 2 | 5 | 0 | 1 |
| 12 | 0 | 1 | 1 | 0 | 0 |
| 13 | 0 | 1 | 1 | 1 | 0 |
| 14 | 0 | 1 | 5 | 13 | 0 |
| 15 | 1 | 1 | 1 | 1 | 5 |
| 16 | 2 | 0 | 0 | 1 | 0 |
| 17 | 1 | 0 | 1 | 1 | 0 |
| 18 | 1 | 1 | 1 | 1 | 0 |
| 19 | 9 | 0 | 0 | 0 | 3 |
| 20 | 1 | 0 | 0 | 4 | 0 |
| 21 | 4 | 0 | 1 | 1 | 0 |
| 22 | 2 | 0 | 0 | 1 | 0 |
| 23 | 0 | 1 | 1 | 1 | 0 |
| 24 | 1 | 1 | 1 | 10 | 0 |
| 25 | 2 | 0 | 0 | 0 | 0 |
| 26 | 7 | 1 | 1 | 0 | 2 |
| 27 | 0 | 2 | 4 | 1 | 5 |
| 28 | 0 | 3 | 0 | 1 | 0 |
| 29 | 1 | 0 | 0 | 1 | 0 |
| 30 | 0 | 1 | 1 | 0 | 0 |
| 31 | 0 | 1 | 1 | 0 | 0 |
| 32 | 0 | 0 | 0 | 0 | 3 |
| 33 | 0 | 1 | 0 | 1 | 0 |
| 34 | 0 | 2 | 0 | 15 | 0 |
| 35 | 0 | 1 | 1 | 0 | 1 |
| 36 | 0 | 1 | 1 | 0 | 2 |
| 37 | 0 | 1 | 1 | 0 | 0 |
| 38 | 1 | 1 | 0 | 1 | 6 |
| 39 | 1 | 0 | 0 | 1 | 0 |
| 40 | 2 | 0 | 0 | 1 | 0 |
| 41 | 0 | 1 | 0 | 2 | 0 |
| 42 | 12 | 0 | 1 | 1 | 0 |
| 43 | 2 | 0 | 0 | 0 | 0 |
| 44 | 0 | 0 | 0 | 1 | 0 |
| 45 | 0 | 1 | 1 | 2 | 0 |
| 46 | 0 | 1 | 1 | 2 | 0 |

**Table S4**. *continuation.*

| Bromeliad number | *An. cruzii* | *An. bellator* | *Cx. imitator* | *Wy. quasilongirostris* | *Wy. muhelensis* |
|---|---|---|---|---|---|
| 47 | 1 | 6 | 7 | 1 | 0 |
| 48 | 1 | 0 | 0 | 0 | 0 |
| 49 | 3 | 1 | 0 | 1 | 0 |
| 50 | 1 | 0 | 0 | 7 | 0 |
| 51 | 0 | 1 | 1 | 0 | 2 |
| 52 | 0 | 0 | 0 | 0 | 6 |
| 53 | 0 | 1 | 1 | 5 | 1 |
| 54 | 0 | 0 | 0 | 0 | 1 |
| 55 | 0 | 1 | 0 | 0 | 1 |
| 56 | 5 | 0 | 0 | 1 | 0 |
| 57 | 1 | 1 | 1 | 0 | 0 |
| 58 | 9 | 0 | 0 | 0 | 2 |
| 59 | 20 | 0 | 0 | 0 | 0 |
| 60 | 1 | 0 | 0 | 2 | 0 |
| 61 | 15 | 0 | 0 | 0 | 2 |
| 62 | 6 | 1 | 1 | 1 | 0 |
| 63 | 0 | 1 | 1 | 0 | 1 |
| 64 | 6 | 0 | 0 | 1 | 1 |
| 65 | 1 | 0 | 0 | 1 | 0 |
| 66 | 0 | 0 | 0 | 1 | 1 |
| 67 | 0 | 1 | 1 | 1 | 0 |
| 68 | 6 | 0 | 0 | 0 | 0 |
| 69 | 1 | 0 | 1 | 0 | 1 |
| 70 | 1 | 1 | 1 | 1 | 1 |
| 71 | 0 | 1 | 1 | 0 | 1 |
| 72 | 0 | 1 | 0 | 2 | 0 |
| 73 | 0 | 1 | 0 | 1 | 0 |
| 74 | 0 | 0 | 0 | 1 | 0 |
| 75 | 0 | 1 | 1 | 0 | 0 |
| 76 | 0 | 0 | 0 | 1 | 0 |
| 77 | 0 | 1 | 0 | 0 | 4 |
| 78 | 0 | 5 | 6 | 0 | 1 |
| 79 | 8 | 1 | 1 | 1 | 0 |
| 80 | 1 | 4 | 0 | 3 | 0 |
| 81 | 0 | 1 | 1 | 1 | 0 |
| 82 | 3 | 0 | 1 | 6 | 1 |
| 83 | 1 | 1 | 1 | 0 | 1 |
| 84 | 1 | 0 | 0 | 0 | 1 |
| 85 | 0 | 1 | 0 | 1 | 0 |
| 86 | 8 | 0 | 0 | 1 | 0 |
| 87 | 0 | 1 | 1 | 3 | 1 |
| 88 | 3 | 0 | 1 | 0 | 1 |
| 89 | 1 | 0 | 0 | 1 | 1 |
| 90 | 1 | 0 | 5 | 0 | 1 |
| 91 | 0 | 0 | 4 | 0 | 0 |
| 92 | 0 | 1 | 1 | 0 | 0 |
| 93 | 0 | 5 | 8 | 1 | 1 |
| 94 | 0 | 0 | 1 | 0 | 0 |

**Table S4**. *continuation.*

| Bromeliad number | *An. cruzii* | *An. bellator* | *Cx. imitator* | *Wy. quasilongirostris* | *Wy. muhelensis* |
|---|---|---|---|---|---|
| 95 | 0 | 1 | 1 | 1 | 0 |
| 96 | 0 | 1 | 0 | 0 | 0 |
| 97 | 0 | 6 | 10 | 0 | 0 |
| 98 | 1 | 1 | 1 | 1 | 0 |

[a]: Bromeliad number 1-33 are from mixed arboreal and scrub vegetation,34-64 are from dense ombrophilous forest and 65-98 are from scrub vegetation.
