# Supplementary material for: Coexistence mechanisms at multiple scales in mosquito assemblages
Source: BMC Ecol. 2014 Nov 11;14:30. doi: 10.1186/s12898-014-0030-8 (PMC4247778; doi:10.1186/s12898-014-0030-8)
Supplement: Additional file 6: Table S5 — Results of the AIC Model Selection approach with mosquito species richness in function of vegetation gradient and its heterogeneity, Parque Estadual da Ilha do Cardoso, 2009-2010. [file 12898_2014_30_MOESM6_ESM.pdf]

Table S5. Results of the AIC Model Selection approach with mosquito species richness in function of vegetation gradient and its heterogeneity, Parque Estadual da Ilha do Cardoso, 2009-2010.

	Models	df[a]	AICc	AICc Weight
**Mosquito richness** *in function of* **vegetation gradient** (Fig. 5A)	Model I (flat)	1	1274.0	0.0
	Model II monotone)	2	1275.8	0.0
	Model III (plateu)	3	1253.0	0.0
	Model IV (symmetric)	3	1035.7	0.0
	Model V (skewed)	4	899.6	0.0
	Model VI (bimodal symmetric)	4	887.3	0.0
	Model VII (bimodal skewed)	5	**824.7**[b]	**1.0**
**Vegetation gradient** *in function of* **vegetation structure** (Fig. 5B)	Model I (flat)	1	554.8	0.0
	Model II (monotone)	2	504.2	0.0
	Model III (plateu)	3	317.3	0.0
	Model IV (symmetric)	3	246.7	0.0
	Model V (skewed)	4	176.3	0.2
	Model VI (bimodal symmetric)	4	187.9	0.0
	Model VII (bimodal skewed)	5	**173.8**	**0.8**
**Mosquito richness** *in function of* **vegetation heterogeneity** (Fig. 5C)	Model I (flat)	1	1274.0	0.0
	Model II (monotone)	2	1070.0	0.0
	Model III (plateu)	3	**1035.4**	**0.8**
	Model IV (symmetric)	3	1046.9	0.0
	Model V (skewed)	4	1037.7	0.2
	Model VI (bimodal symmetric)	4	1049.6	0.0
	Model VII (bimodal skewed)	5	1052.5	0.0

[a]: The number of parameters in the Model Selection varies, accordingly: flat, one parameter; monotone, two parameters; plateau, three parameters; symmetric, three parameters; skewed, four parameters; bimodal symmetric, four parameters and bimodal skewed, five parameters.

[b]: The results printed in bold represent the most plausible model under the lowest value of Akaike Information Criteria based on the best model's parsimony and likelihood [34].
