# Supplementary figures and images for: Coexistence mechanisms at multiple scales in mosquito assemblages
Source: BMC Ecol. 2014 Nov 11;14:30. doi: 10.1186/s12898-014-0030-8 (PMC4247778; doi:10.1186/s12898-014-0030-8)

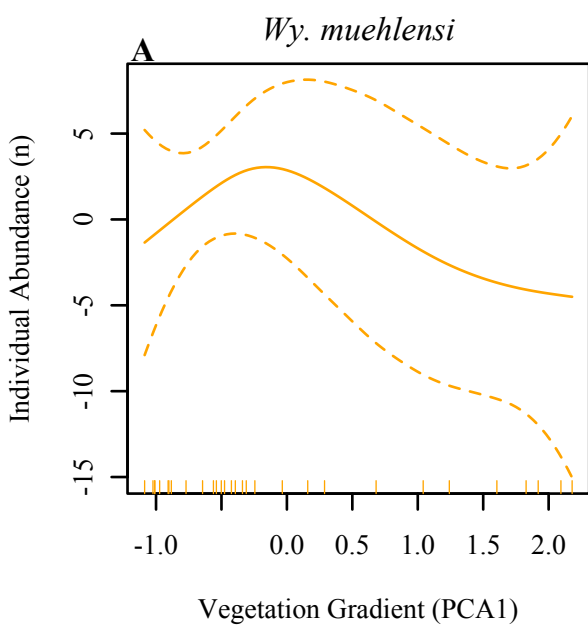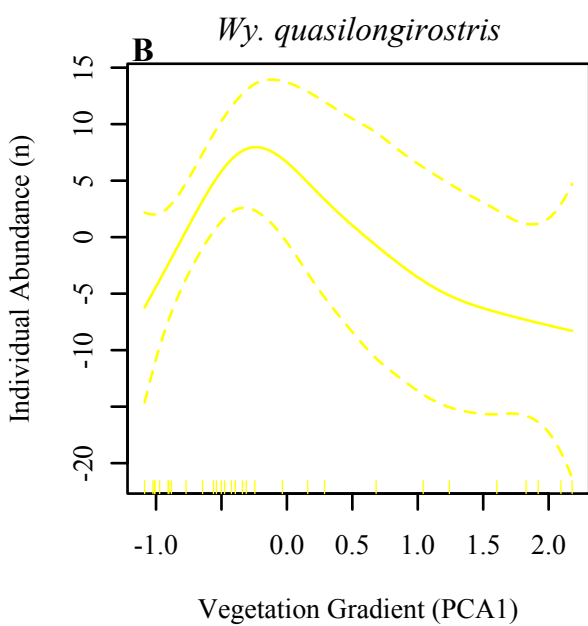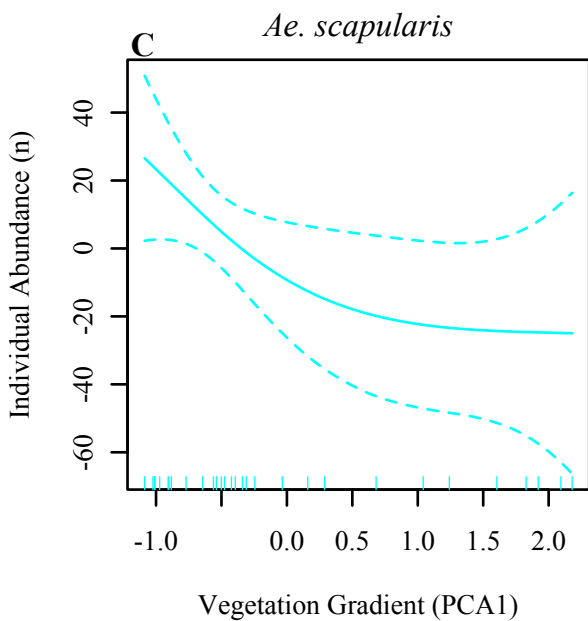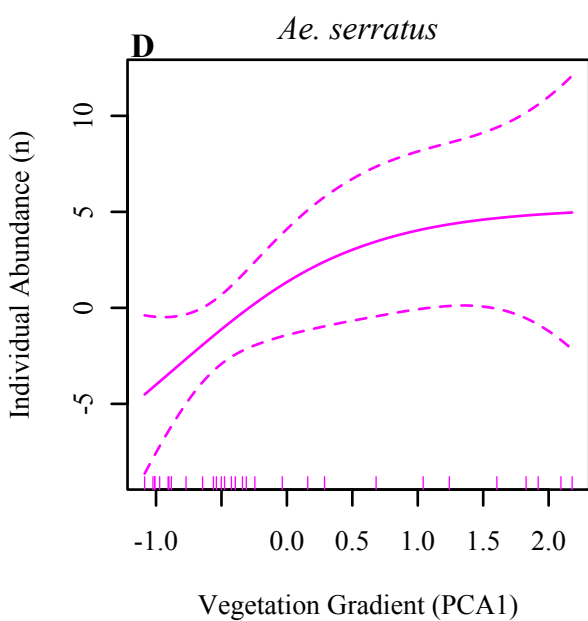

Supplement: Additional file 7: Figure S5 — Solid lines represent GAM fitted to the data using cubic regression splines. Dashed lines define the 95% CI. A) Figure 4B-Wy. muhelensi. B) Figure 4B-Wy. quasilongirostris. C) Figure 4D-Ae. scapularis. D) Figure 4D-Ae. serratus. [file 12898_2014_30_MOESM7_ESM.pdf]

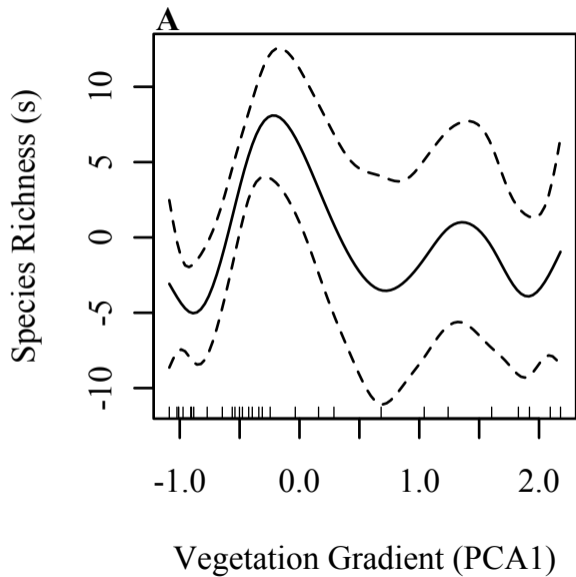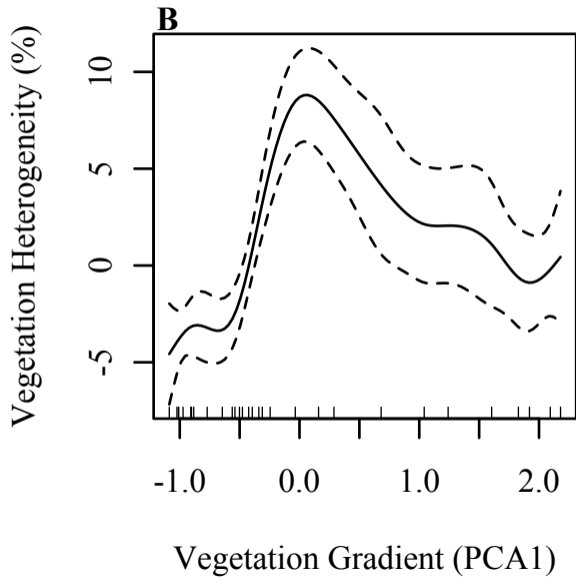

Supplement: Additional file 8: Figure S6 — Solid lines represent GAM fitted to the data using cubic regression splines. Dashed lines define the 95% CI. A) Figure 5A. B) Figure 5B. [file 12898_2014_30_MOESM8_ESM.pdf]

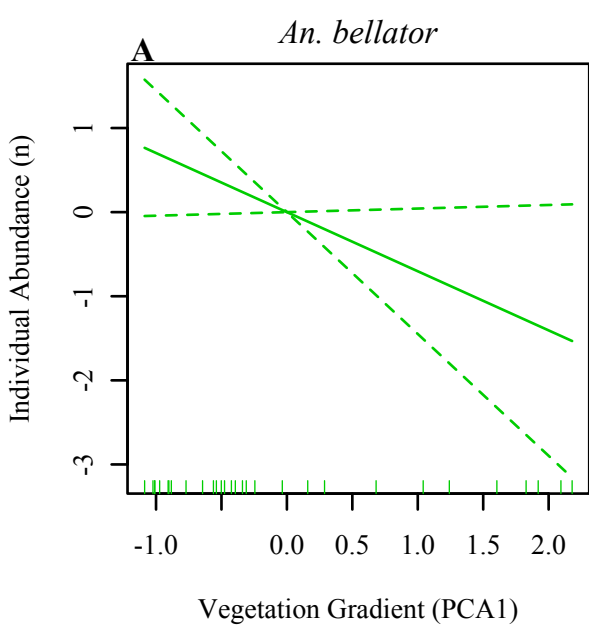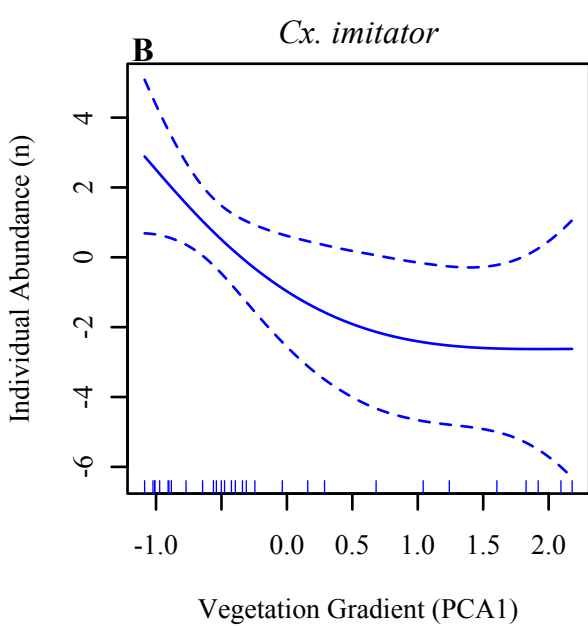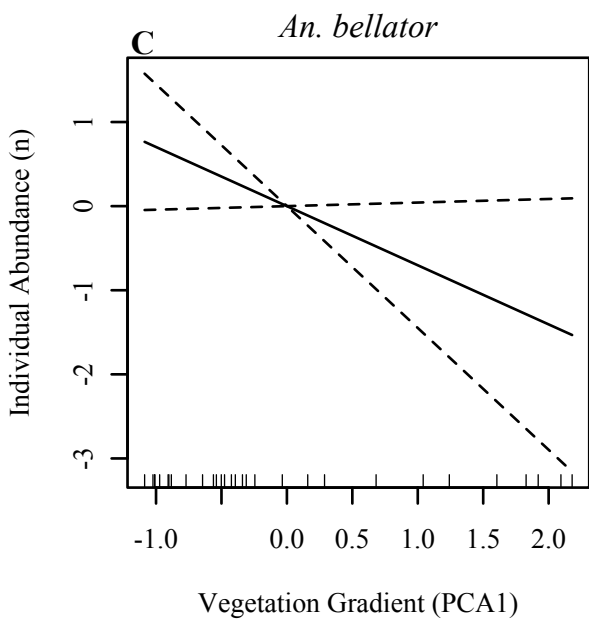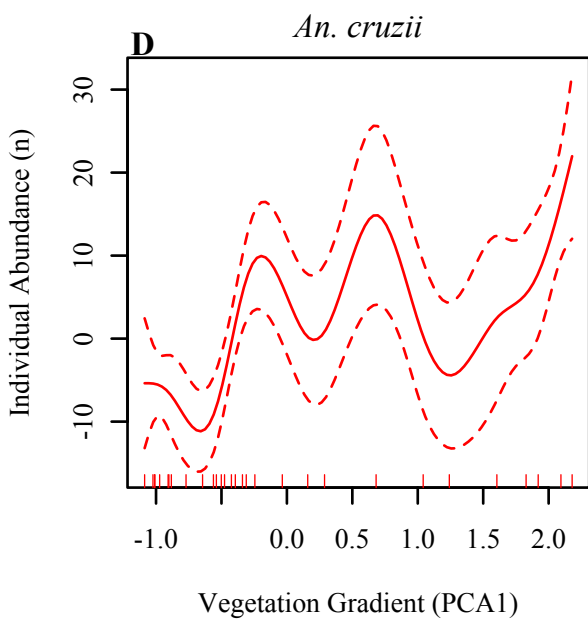

Supplement: Additional file 9: Figure S7 — Solid lines represent GAM fitted to the data using cubic regression splines. Dashed lines define the 95% CI. A) Figure 4A-An. bellator. B) Figure 4A-Cx. imitator. C) Figure 4C-An. bellator. D) Figure 4C-An. cruzii. [file 12898_2014_30_MOESM9_ESM.pdf]
